# Effectiveness of Entomopathogenic Fungi on Immature Stages and Feeding Performance of Fall Armyworm, *Spodoptera frugiperda* (Lepidoptera: Noctuidae) Larvae

**DOI:** 10.3390/insects12111044

**Published:** 2021-11-21

**Authors:** Atif Idrees, Ziyad Abdul Qadir, Komivi Senyo Akutse, Ayesha Afzal, Mubasher Hussain, Waqar Islam, Muhammad Saad Waqas, Bamisope Steve Bamisile, Jun Li

**Affiliations:** 1 Guangdong Key Laboratory of Animal Conservation and Resource Utilization, Guangdong Public Laboratory of Wild Animal Conservation and Utilization, Institute of Zoology, Guangdong Academy of Sciences, Guangzhou 510260, China; atif_entomologist@yahoo.com; 2 Honeybee Research Institute, National Agricultural Research Centre, Park Road, Islamabad 45500, Pakistan; zaqadir@parc.gov.pk; 3 Department of Entomology and Wildlife Ecology, University of Delaware, Newark, DE 19716, USA; 4 Plant Health Theme, International Centre of Insect Physiology and Ecology, Nairobi P.O. Box 30772-00100, Kenya; kakutse@icipe.org; 5 Institute of Molecular Biology and Biotechnology, The University of Lahore, 1-Km Defense Road, Lahore 54000, Pakistan; ayeshaafzal403@yahoo.com; 6 Guangdong Key Laboratory of Animal Conservation and Resource Utilization, Guangdong Public Laboratory of Wild Animal Conservation and Utilization, Guangdong Engineering Research Center for Mineral oil pesticides, Institute of Zoology, Guangdong Academy of Science, Guangzhou 510260, China; mubasherhussain05uaf@yahoo.com; 7 Xinjiang Institute of Ecology and Geography, Chinese Academy of Sciences, Urumqi 830011, China; ddoapsial@yahoo.com; 8 University of Chinese Academy of Sciences, Beijing 100049, China; 9 Provincial Key Laboratory for Agricultural Pest Management of Mountainous Regions, and Scientific Observing and Experimental Station of Crop Pest in Guiyang, Institute of Entomology, Ministry of Agricultural and Rural Affairs, Guizhou University, Guiyang 550025, China; muhammadsaad@zju.edu.cn; 10 Laboratory of Quarantine and Invasive Pests, Department of Entomology, South China Agricultural University, Guangzhou 510642, China; bamisopebamisile@gmail.com

**Keywords:** entomopathogens, immature stages, feeding ability, egg hatchability, *Spodoptera frugiperda*

## Abstract

**Simple Summary:**

Fall armyworm (FAW), primarily endemic to the United States, has posed a severe threat to maize cultivation globally in recent decades. To prevent maize from being harmed by FAW, various control strategies are used, including synthetic pesticides. Synthetic chemicals are still the most effective and widely utilized technique; nonetheless, these chemicals are hazardous to humans, biodiversity, and the environment, necessitating a desperate search for safe and long-term solutions. Entomopathogenic fungi (EPFs) are thought to be an essential alternate control tool for this invasive pest. The goal of this study was to determine the effectiveness of five entomopathogenic fungal isolates (*Aspergillus* sp. BM-3 and SE-2-1, *Cladosporium tenuissimum* SE-10, *Penicillium citrinum* CTD-24, and *Beauveria bassiana* ZK-5) against immature stages (eggs, neonates, and larvae) and feeding efficacy of first to sixth instar *S. frugiperda* larvae at 1 × 10^6^, 1 × 10^7^, and 1 × 10^8^ conidia mL^−1^. Among the five tested fungal isolates, *C. tenuissimum* SE-10, *P. citrinum* CTD-24, and *B. bassiana* ZK-5 showed significant effects on egg mortality and significantly reduced the early third instar feeding efficacy of FAW larvae at the highest concentration level. These potent fungal isolates could be suitable candidates for developing biopesticides in an integrated manner to control the FAW population. By decreasing the hatchability of eggs and reducing the feeding ability of early first to third instar FAW larvae, the findings of this study could assist in managing this invasive pest in China and enhance maize crop output. However, further research is needed to evaluate and validate laboratory outcomes in real-world situations.

**Abstract:**

Maize is a major staple crop in China, and the sustainable productivity of this primary crop has been recently threatened by fall armyworm (FAW), *Spodoptera frugiperda*, invasion. The five fungal isolates, *Aspergillus* sp. BM-3 and SE-2-1, *Cladosporium tenuissimum* SE-10, *Penicillium citrinum* CTD-24, and *Beauveria bassiana* ZK-5 were assessed for their efficacy in causing mortality against first to sixth instar eggs and neonate larvae seven days post-treatment, and their effects on the feeding performance of sixth instar *S. frugiperda* larvae at 48 h post-treatment at three concentrations (1 × 10^6^, 1 × 10^7^, and 1 × 10^8^ conidia mL^−1^) were also assessed. The six instar *S. frugiperda* larvae were not susceptible to the five tested fungal isolates. However, *B. bassiana* ZK-5 caused the highest egg mortality of 40, 70, and 85.6% at 1 × 10^6^, 1 × 10^7^, and 1 × 10^8^ conidia mL^−1^, respectively, followed by *P. citrinum* CTD-24 (30.6, 50, and 75.6%) and *C. tenuissimum* SE-10 (25.6, 40, and 55.6%). In addition, *B. bassiana* ZK-5 caused the highest neonate mortality of 54.3% at 1 × 10^8^ conidia mL^−1^. *B. bassiana* ZK-5 and *P. citrinum* CTD-24 caused cumulative mortality, including 93.3 and 83.3% mortality of eggs and neonates, respectively, at 1 × 10^8^ conidia mL^−1^. Furthermore, *B. bassiana* ZK-5 reduced the feeding efficacy of first to third instar *S. frugiperda* larvae by 66.7 to 78.6%, while *P. citrinum* CTD-24 and *C. tenuissimum* SE-10 reduced larval feeding by 48.3 to 57.1% at 1 × 10^8^ conidia mL^−1^. However, these fungal isolates were less potent in reducing the feeding activity of fourth to sixth instar *S. frugiperda* larvae (>46% with *B. bassiana* at 48 h post-treatment). The tested fungal isolates could play an essential role as microbial biopesticides in suppressing the *S. frugiperda* population in China after further investigations on their efficacy are obtained in the field.

## 1. Introduction

Maize is a significant crop in China, especially since the country has become a net importer of grain [1]. China is the world’s second-largest corn producer after the U.S. However, maize production has been threatened by fall armyworm (FAW), *Spodoptera frugiperda*, J.E. Smith, 1797, (Lepidoptera: Noctuidae), which is highly ranked as one of the most destructive pests of the maize crop. This pest is native to the American continent, including tropical and subtropical regions [2], and has recently invaded Africa, Asia, and Australia with a highly detrimental impact on global food security in all invaded areas. FAW was first reported in West Africa (Nigeria and Ghana) [3] and later spread to other countries in sub-Saharan Africa [4]. In May 2018, FAW was observed in Karnataka in the southwestern part of India [5], and its occurrence was also reported in southeastern Asian countries, such as Thailand, Bangladesh, and Myanmar, by late 2018 [6].

FAW was first reported in China on 11 December 2018 [7], when the corn-specific strain of *S. frugiperda* invaded [8]. *Spodoptera frugiperda* invaded 26 provinces after migrating to China from Myanmar, and Guangxi and Yunnan were the major areas invaded by the pest [9]. The larvae of *S. frugiperda* have the potential to damage over 350 species of plants, including major staple and economic crops such as sorghum, corn, barley, rice, soybean, tobacco, tomato, and peanut [10], but show a high preference for corn, sugarcane, and sorghum in China. During the early attack of *S. frugiperda* larvae in 2019, extremely significant damage was caused to cornfields in Yunnan Province, China [11]. *Spodoptera frugiperda* was reported to cause 67 and 22% maize yield losses in Zambia and Ghana, respectively [12], while in Kenya and Ethiopia, this invasive pest caused maize yield losses of 47 and 32%, respectively [13], resulting in economic losses of 1.08 and 4.66 billion USD in Africa [14]. *Spodoptera frugiperda* larvae were also reported to feed on growing points and young organs in the peanut field and cause 78 and 65% damage at seedling and flowering stages, respectively [15]. *Spodoptera frugiperda* causes serious damage (30 to 90%) in wheat fields, while 10 to 80% damage was recorded in the barley field [16].

This invasive pest can potentially cause damage to tobacco fields if its population reaches its peak [17]. Therefore, many studies have focused on developing biopesticides as a component of integrated pest management (IPM) against insect pests, to minimize damage and increase the productivity of crops [18,19,20,21,22,23]. However, continuous applications of synthetic insecticides by farmers to control *S. frugiperda* have become a common practice, which is not only uneconomical but also harmful to the environment and natural enemies [24,25] and consequently leads to the development of pest resistance. Some pesticides, e.g., methomyl, endosulfan, methyl parathion, and lindane, have been used for *S. frugiperda* management and are known to be highly hazardous [26,27], and endosulfan and lindane are banned in many countries [28]. Moreover, pesticides are not safe for farmers due to their toxicity, and the majority of farmers have little information or knowledge on the precautionary measures to take during the application of these insecticides in the field. Hence, there is a strong need to develop alternative management methods to synthetic insecticides that are safe, environmentally friendly, and economical, to sustainably control *S. frugiperda*.

Entomopathogenic fungi (EPFs) have been recognized as comprehensive biopesticides in the management of many destructive pests, and could also be integrated as a key component of IPM strategies for FAW control [29,30] as they were found to be an efficient control practice against a variety of insect pests [31,32]. One of the perceived disadvantages of EPFs is that they do not cause quick mortality (killing speed) to insects, similar to their counterpart synthetic insecticides, due to their different modes of action, therefore insects continue to feed and cause damage to crops [33,34]. However, it is important to note that these EPFs help to minimize damage to crops (below the economic threshold) by inducing infection of host pests, which ultimately leads to a reduction in feeding, oviposition, development, mating, and other physiological traits of insects [35]. A recent study revealed that some *Metarhizium anisopliae* isolates showed significant mortality to *S. frugiperda* early immature stages [36].

However, there is little evidence on the efficiency of locally available EPFs in China that are associated with eggs and can affect the feeding performance of *S. frugiperda* larvae. Hence, the objective of this work was to find effective fungal isolates that can disrupt the eggs and feeding of *S. frugiperda*, to develop microbial-based biopesticides for use against this notorious invasive lepidopteran pest in China and abroad.

## 2. Materials and Methods

### 2.1. Insect Culture

*Spodoptera frugiperda* eggs were obtained from the already established colony at the Institute of Zoology, Guangdong Academy of Sciences, Guangdong, Guangzhou, China. Eggs were placed in a perforated rectangular plastic box (28 × 17 × 18 cm^3^) and monitored daily until hatching. After emergence, neonate larvae were given tender and fresh maize leaves. First to third instar larvae were supplied with fresh maize leaves daily in a perforated rectangular plastic box (28 × 17 × 18 cm^3^), while fourth to sixth instar larvae were individually placed in wells in a six-well plate to avoid cannibalism until pupation. Male and female pupae (50:50) were collected and placed in a perforated rectangular plastic box (28 × 17 × 18 cm^3^) until adult emergence.

The newly emerged adults were transferred into adult glass cylindrical cages. The upper portion of the adult cylindrical glass cage was covered with a paper towel, while the inner wall of the adult cage was covered with white paper serving as an oviposition medium. Sterile cotton balls were placed in a plastic bottle lid soaked with 10% honey solution, and then the lid was placed at the bottom of the adult cage as a food source. The paper towel, white paper, and sterile cotton ball were replaced every 24 h to ensure homogenous egg ages. Insects were reared as described above until a sufficient population was achieved to conduct the experiment. Tenth-generation, laboratory-reared larvae were used for the present study. *Spodoptera frugiperda* colonies were maintained in a rearing room at 25 ± 2 °C, 50–70% relative humidity (RH), and an 18:6 (Dark:Light) photoperiod at the Institute of Zoology, Guangdong Academy of Sciences.

### 2.2. Fungal Isolates

Five fungal isolates belonging to four different genera, *Aspergillus* sp. (BM-3 and SE-2-1), *Cladosporium tenuissimum* SE-10, *Penicillium citrinum* CTD-24, and *Beauveria bassiana* ZK-5, were assessed against eggs and sixth instar, *S. frugiperda* larvae in terms of feeding performance. Information regarding their origin, host source, and year of isolation is described in Table 1. All fungal isolates were obtained from the Institute of Zoology, Guangdong Academy of Sciences, laboratory repository. All fungal isolates were cultured on potato dextrose agar (PDA) and maintained at 25 ± 2 °C in the dark.

Fungal conidia were harvested from 2- to 3-week-old sporulated cultures and suspended in 10 mL of distilled water with 0.05% Tween-80 in universal bottles containing glass beads (6–9 beads 3 mm in diameter per bottle). The fungal conidial suspension was vortexed for 5 min at approximately 700 rpm to break the conidial clumps and ensure a homogeneous suspension. Conidial concentrations were quantified using a hemocytometer. Before the bioassay, the conidial suspensions were adjusted to different concentrations of 1 × 10^6^, 1 × 10^7^, and 1 × 10^8^ conidia mL^−1^ through serial dilution.

Viability tests were conducted before experiments, where a concentration of 3 × 10^6^ conidia mL^−1^ was prepared, 0.1 mL of the suspension was evenly spread on a PDA plate, and three sterile microscope coverslips were placed randomly on the surface of each inoculated plate. Each plate represented a replicate, and four replicates were performed for each isolate. The plates were sealed with parafilm and incubated under complete darkness at 25 ± 2 °C. Conidial germination was assessed after 18 h by counting 100 randomly selected conidia beneath each coverslip, under a light microscope [37,38]. Conidia were considered germinated when the length of the germ tube was at least twice the diameter of the conidium [39,40]. The viability of each of the fungal isolates was assessed (Table 2).

### 2.3. Effect of Fungal Isolates on Sixth Instar S. frugiperda Larvae

All five fungal isolates were assessed against sixth instar *S. frugiperda* larvae obtained from an already established insect colony, as described in Section 2.1 above. Fresh maize leaves of similar size and age, approximately 2–3 weeks old, were cut and placed individually in a perforated rectangular plastic box (28 × 17 × 18 cm^3^). A cohort of 20 first instar *S. frugiperda* larvae was transferred onto maize leaves in each rectangular plastic box using a soft camel hairbrush. Each group of first instar larvae in a rectangular plastic box represented a replicate, and triplicate groups were analyzed for each isolate. The cut leaves with larvae in each rectangular plastic box were then sprayed with 10 mL of 1 × 10^6^, 1 × 10^7^, or 1 × 10^8^ conidia mL^−1^ suspension using a spray tower. All the larvae were effectively exposed to the fungal spore suspension to prevent larval escape to the inoculum by going beneath the leaves. A sterilized paper towel was placed under the leaves to absorb the excess fungal suspension before applying the spray. The control was treated with sterile distilled water containing 0.05% Tween-80. The rectangular plastic boxes containing treated larvae were incubated at 25 ± 2 °C. Fresh maize leaves (surface-sterilized and untreated) were provided to the larvae daily as food. Larval mortality was recorded daily for 1 week [38]. All the treatments were arranged in a completely randomized design (CRD), with each treatment conducted in triplicate. A mycosis test was conducted following the approach of [36] to confirm larval mortality due to infection by the treated fungus. The same procedure was followed for second to sixth instar *S. frugiperda* larvae, while fourth to sixth instar larvae were placed individually in a six-well plate before treatment, to avoid cannibalism.

### 2.4. Effect of Fungal Isolates on Eggs and Neonate Larvae of S. frugiperda

The eggs (1 to 2 days old) of *S. frugiperda* laid on paper towels and white paper, as described in Section 2.1 above, were collected from the adult cages. A cohort of 30 eggs per butter paper was separated under the microscope by removing excess eggs with a camel hairbrush before treatments. The cohort of eggs was treated with 10 mL of 1 × 10^6^, 1 × 10^7^, and 1 × 10^8^ conidia mL^−1^ suspensions of each fungal isolate by a spray tower, where a sterilized paper towel was placed under the butter paper to absorb the excess spore suspension. The controls were treated with sterile distilled water containing 0.05% Tween-80. After treatment, eggs were air-dried for 1 h under a laminar hood at room temperature (25 ± 2 °C), transferred into Petri dishes, and incubated at 25 ± 2 °C and an approximate RH of 70%.

All the treatments were arranged in a CRD with three replications per fungal isolate and concentration. The numbers of hatched and non-hatched eggs were recorded daily until 7 days after incubation. The neonate larvae that emerged from the treated egg cohort were counted, fed fresh maize leaves (approximately 2–3 weeks old, surface-sterilized, and untreated) in a perforated rectangular plastic box (28 × 17 × 18 cm^3^) lined with moist filter paper, incubated at 25 ± 2 °C, and monitored daily. Neonate mortality due to fungal infection was recorded daily for 7 days postemergence, and a mycosis test was also conducted for the dead neonates, as described in Section 2.3 above.

### 2.5. Effect of Fungal Isolates on Feeding Performance of Sixth Instar S. frugiperda Larvae

Fresh maize leaves were collected separately and placed in a perforated rectangular plastic box (28 × 17 × 18 cm^3^). The collected leaves were cut and weighed to the required quantity (45–60 g of maize leaves can feed approximately 20 to 25 larvae for 2–3 days, from our *S. frugiperda*-rearing experience). The leaves were placed in a rectangular box. The larvae were treated with each fungal isolate with 10 mL of 1 × 10^6^, 1 × 10^7^, or 1 × 10^8^ conidia mL^−1^ suspension using a spray tower [36,41]. Twenty-five first instar *S. frugiperda* larvae were released in a rectangular plastic box containing treated leaves. A quantity of 7, 12, 20, 30, 45, and 60 g of maize leaves were provided daily to first, second, third, fourth, fifth, and sixth instar larvae, respectively. Larvae treated with sterile distilled water containing 0.05% Tween-80 were considered controls. The treatments were laid out in a CRD with triplicate samples. The insect diet (weighed maize leaves) was changed every second day post-treatment. The leaves were weighed before and after exposure to fungal isolates to assess the feeding performance of *S*. *frugiperda* larvae.

### 2.6. Statistical Analysis

Egg and larval mortality data were corrected using Abbott’s formula [42] to correct for natural mortality, and their normality was tested using the Shapiro–Wilk test [43] before being subjected to one-way analyses of variance (ANOVA). In the case where the data were not normally distributed, they were further arcsine transformed before analysis. Whenever treatments were significantly different (*p* < 0.05), the means were separated using the Tukey test. All data analyses, including a reduction in the feeding of sixth instar *S. frugiperda* larvae, were performed using SPSS (version 16.0), and letters were calculated using Statistix 8.1 software.

## 3. Results

### 3.1. Effect of Fungal Isolates on Sixth Instar of S. frugiperda Larvae

The viability of the five tested fungal isolates was >91% conidial germination after 18 h of incubation at 25 ± 2 °C, as shown in Table 2, with no significant differences among the isolates.

The results revealed no significant differences between all the tested fungal isolates against first to sixth instar *S. frugiperda* larvae 7 days post-treatment. Larval mortality was below 6% for *B. bassiana* isolate exposure at 1 × 10^6^ conidia mL^−1^ (first instar: F = 1.56; df = 17; *p* > 0.05; sixth instar: F = 1.60; df = 17; *p* > 0.05) against first and sixth instar *S. frugiperda* larvae. *B. bassiana* caused larval mortality ranging between 14.4 and 5.6% with 1 × 10^7^ conidia mL^−1^ (first instar: F = 1.06; df = 17; *p* > 0.05; sixth instar: F = 2.51; df = 17; *p >* 0.05) against first and sixth instar *S. frugiperda* larvae. The highest larval mortality, ranging between 20.0 and 7.8% for *B. bassiana,* was only observed at 1 × 10^8^ conidia mL^−1^ (first instar: F = 18.7; df = 17; *p* < 0.05; sixth instar: F = 1.50; df = 17; *p >* 0.05) against first- to sixth-instar *S. frugiperda* larvae. No significant differences compared to the control were observed for any fungal isolates at any concentration level (Table 3). Furthermore, mycosis was only observed in 10% of the dead insects.

### 3.2. Effect of Fungal Isolates on Eggs of S. frugiperda

**Figure 1:**
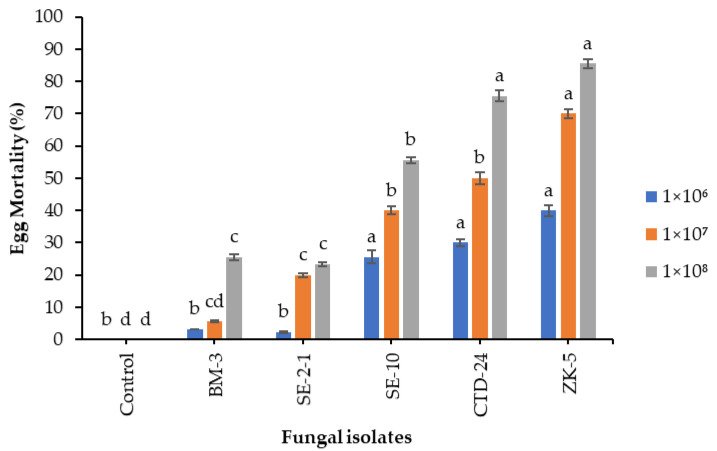
Effects of fungal isolates, i.e., *Aspergillus* sp. BM-3 and SE-2-1, *C. tenuissimum* SE-10, *P. citrinum* CTD-24, and *B. bassiana* ZK-5 on *S. frugiperda* egg mortality. Means followed by the same letters are not significantly different at *p* < 0.05 using Tukey’s test.

The results revealed significantly different impacts of the fungal isolates on egg hatchability, leading to varied mortality rates of *S. frugiperda* eggs 7 days post-treatment with 1 × 10^6^ conidia mL^−1^ (F = 19.4; df = 20; *p* < 0.0000). *Beauveria bassiana* ZK-5 and *P. citrinum* CTD-24 isolates caused egg mortalities of 40 and 30.6%, respectively, followed by *C. tenuissimum* SE-10 (25.6%). However, no significant differences compared to the controls were observed with *Aspergillus* sp. (BM-3 and SE-2-1) isolates at the same concentration of 1 × 10^6^ conidia mL^−1^. *Beauveria bassiana* ZK-5, *P. citrinum* CTD-24, and *C. tenuissimum* SE-10 isolates caused significant egg mortalities of 70, 50, and 40%, respectively, at 1 × 10^7^ conidia mL^−1^ (F = 57.6; df = 20; *p* < 0.0000), compared to 0.0% in the control. However, *Aspergillus* sp. isolates BM-3 and SE-2-1 did not cause mortalities that were significantly different from that of the control. At a concentration of 1 × 10^8^ conidia mL^−1^, *B. bassiana* ZK-5, *P. citrinum* CTD-24, and *C. tenuissimum* SE-10 isolates outperformed the other isolates (F = 83.2; df = 20; *p* < 0.0000), causing the highest egg mortalities of 85.6, 75.6, and 55.6%, respectively, while no significant difference was observed between *Aspergillus* sp. isolates BM-3 and SE-2-1, that caused only 23.3 and 25.6% egg mortalities, respectively (Figure 1).

### 3.3. Effect of Fungal Isolates on Neonate Larvae of S. frugiperda

**Figure 2:**
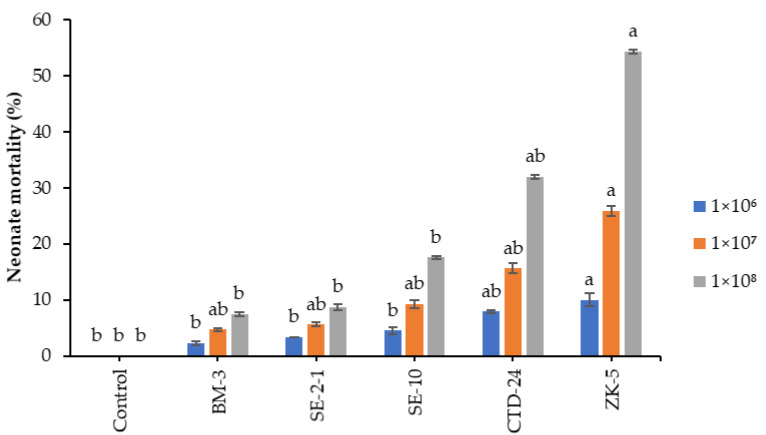
Fungal isolates, i.e., *Aspergillus* sp. BM-3 and SE-2-1, *C. tenuissimum* SE-10, *P. citrinum* CTD-24, and *B. bassiana* ZK-5-induced mortalities of the newly emerged *S. frugiperda* larvae. Means followed by the same letters are not significantly different at *p* < 0.05 using Tukey’s test.

In addition to egg mortality, the fungal isolates induced additional mortality of the neonate larvae that emerged from the fungus-treated *S. frugiperda* eggs compared to the control 7 days postemergence. The neonate larvae were found to be susceptible to all the fungal isolates at 1 × 10^6^ conidia mL^−1^ (F = 5.08; df = 20; *p* < 0.0058), except *B. bassiana* ZK-5, which caused only 10% neonate mortality compared to that of the control (0.0%) (Figure 2; Table 4).

At a concentration of 1×10^7^ conidia mL^−1^, *B. bassiana* ZK-5 and *P. citrinum* CTD-24 isolates caused higher neonate mortalities of 25.9 and 15.6%, respectively (F = 3.81; df = 20; *p* < 0.0185), than the 0% in the control. However, no significant differences in mortality were observed among other fungal isolates compared to the control (Figure 2; Table 5).

At a concentration of 1 × 10^8^ conidia mL^−1^, *B. bassiana* ZK-5, and *P. citrinum* CTD-24 isolates caused significant (F = 5.78; df = 20; *p* < 0.0033) mortality rates of 54.3 and 32%, respectively, compared to the 0.0% mortality of the control (Figure 2; Table 6). However, no significant differences in mortality were observed between the control and the rest of the fungal isolates. Furthermore, mycosis of the dead neonates was only observed in 5% of insect cadavers.

### 3.4. Effect of Fungal Isolates on Cumulative Mortality of Eggs and Neonates of S. frugiperda

The fungal isolates had significant differences in the cumulative mortality rates of egg and neonate larvae 14 days post-treatment at 1 × 10^6^ conidia mL^−1^ (F = 33.2; df = 20; *p* < 0.0000). *Beauveria bassiana* ZK-5 and *P. citrinum* CTD-24 isolates caused cumulative mortalities of 52.2 and 37.8%, respectively, followed by *C. tenuissimum* SE-10 (32.2%), compared to the 0.0% mortality of the control. However, no significant difference in mortality was observed between *Aspergillus* sp. isolates BM-3 and SE-2-1 and the control (Table 4).

At a concentration of 1 × 10^7^ conidia mL^−1^, *B. bassiana* ZK-5 and *P. citrinum* CTD-24 isolates caused significantly high mortalities of 73.3 and 53.3%, respectively (F = 58.9; df = 20; *p* < 0.0000), followed by *C. tenuissimum* SE-10 (43.3%), while *Aspergillus* sp. BM-3 did not show any significant variation in mortality compared to that of the control (Table 5).

Significant differences were observed among the mortalities caused by fungal isolates at 1 × 10^8^ conidia mL^−1^ (F = 91.1; df = 20; *p* < 0.0000), where *B. bassiana* ZK-5 and *P. citrinum* CTD-24 isolates outperformed all the other fungal isolates by causing the highest cumulative mortalities of 93.3 and 83.3%, respectively, followed by *C. tenuissimum* SE-10 (63.3%). In contrast, the *Aspergillus* sp. isolates caused ~30% cumulative egg and neonate mortalities (Table 6).

### 3.5. Effect of Fungal Isolates on Feeding Performance of Sixth Instar S. frugiperda Larvae

The fungal isolates were least effective in reducing the feeding ability of first to sixth instar *S. frugiperda* larvae at 1 × 10^6^ conidia mL^−1^. *Beauveria bassiana* ZK-5, *P. citrinum* CTD-24, and *C. tenuissimum* SE-10 reduced the feeding efficacy of first to third instar larvae by 33.3–29.2%, 23.8–20.8%, and 19–15%, respectively, while the feeding efficacy of fourth to sixth instar *S. frugiperda* larvae was reduced by 12.8, 11.7, and 9.4%, respectively. However, no significant differences were observed among *Aspergillus* sp. isolates SE-2-1 and BM-3 and the control for first to sixth instar *S. frugiperda* larvae 48 h post-treatment at the same concentration of 1 × 10^6^ conidia mL^−1^ (Table 7).

There was a significant difference among fungal isolates in reducing the feeding performance of first to sixth instar *S. frugiperda* larvae at 1 × 10^7^ conidia mL^−1^ 48 h posttreatment. *Beauveria bassiana* ZK-5, *P. citrinum* CTD-24 and *C. tenuissimum* SE-10 fungal isolates reduced the feeding ability of first to third instar *S. frugiperda* larvae by 59.5–50.8%, 47.6–42.5%, and 42.9–37.5%, respectively, while the feeding was reduced by 31.1–21.7%, 22.8–18.6%, and 20.0–14.2% for fourth to sixth instar *S. frugiperda* larvae, respectively. However, no significant differences were observed for feeding between the *Aspergillus* sp. SE-2-1 and BM-3 isolates and control (Table 8).

At a concentration of 1 × 10^8^ conidia mL^−1^, a significant difference was observed among fungal isolates in reducing the feeding performance of first to sixth instar *S. frugiperda* larvae at 48 h post-treatment (Table 8). *Beauveria bassiana* ZK-5, *P. citrinum* CTD-24, and *C. tenuissimum* SE-10 significantly reduced the feeding performance of first to third instar *S. frugiperda* larvae by 78.6–66.7%, 57.1–48.3%, and 54.8–45%, respectively, while the feeding was reduced by 46.1–38.6%, 37.2–22.5%, and 39.4–21.9% for fourth to sixth instar *S. frugiperda* larvae, respectively. Similarly, *Aspergillus* sp. isolates SE-2-1 and BM-3 induced feeding ability of first to third instars by 33.3–25.8% and 21.4–17.5%, respectively, while they reduced the feeding of fourth to sixth instars of *S. frugiperda* larvae by 23.3–13.6%, and 12.2–6.1%, respectively, at 48 h post-treatment (Table 9).

## 4. Discussion

Numerous pathogens, including viruses, fungi, protozoa, nematodes, and bacteria, have been associated with *S. frugiperda* [44], but only a few cause disease to the pest [45,46,47,48,49,50,51]. *Spodoptera frugiperda* nuclear polyhedrosis virus (NPV) is the most important among these pathogens and induces significant mortality to the pest and the entomopathogenic fungal species *Erynia radicans*, *Nomuraea rileyi,* and *Entomophaga aulicae*. Disease or natural epizooty mostly appear too late in the pest population to alleviate high levels of defoliation, regardless of causing microbial agents. Thus, any entomopathogens that might cause high infection to immature stages (eggs and neonate larvae) of *S. frugiperda,* before it reaches its voracious feeding stage, could significantly suppress the pest populations. Therefore, the present study focused on screening selected entomopathogenic fungal isolates that could effectively induce high infections to immature stages of *S. frugiperda,* as a prerequisite for developing biopesticides to use against the pest.

The results indicated that first to sixth instar *S. frugiperda* larvae were not susceptible to the tested entomopathogenic fungal isolates at different concentrations. Previous studies reported that even though some fungal isolates, e.g., *Aspergillus* sp., were found to be least virulent, *B. bassiana* was the most virulent to the fourth instar striped rice stem borer larvae, *Chilo suppressalis* (Walker) (Lepidoptera: Crambidae) [52], while another study reported that *B. bassiana* caused 12.99% larval mortality to *S. frugiperda* [53]. Similarly, although *P. citrinum* did not cause significant larval mortality to *S. frugiperda*, it was identified as the most toxic isolate, causing 98.67% mortality to the second instar cotton leafworm larvae, *Spodoptera litura* (Fabricius) (Lepidoptera: Noctuidae) [54]. *Penicillium citrinum* strains are also known to be associated with mosquito larvae [55], causing 100% mortality to the third instar southern house mosquito larvae, *Culex quinquefasciatus* (Say) (Diptera: Culicidae), within 2 h, using a conidial suspension at a concentration of 1 *×* 10^6^ conidia mL^−1^ in a laboratory experiment [56]. *Beauveria bassiana* isolates caused 3.3–88.5% mortality to second instar larvae of the spotted stem borer, *Chilo partellus* (Swinhoe) (Lepidoptera: Pyralidae), while 30–84.4% mortality was obtained against fifth and sixth instar larvae of the maize stalk borer, *Busseola fusca* (Fuller) (Lepidoptera: Noctuidae) [57]. In previous studies, some fungal isolates showed high virulence to first instar larvae, while low virulence to later instar larvae was observed; for instance, *Cladosporium* sp. caused 54% mortality in first instar larvae but was least pathogenic to later instar of the corn earworm larvae, *Helicoverpa armigera* (Hübner) (Lepidoptera: Noctuidae) [58]. Few species in the genus *Cladosporium* have been reported to be pathogenic against homopteran insects such as aphids [59] and whiteflies [60]. Second instar *S. frugiperda* larvae were also reported to be less susceptible to the *B. bassiana* ICIPE 676 isolate, which caused moderate larval mortality of 30% [36]. However, *B. bassiana* isolates from soil caused 98.3% mortality to third instar *S. frugiperda* larvae, while fungi of the same strain, isolated from endophytically colonized maize plants, caused 75% mortality to third instar *S. frugiperda* larvae [61]. Variations in the effectiveness of *B. bassiana* against different instars of *S. frugiperda* larvae have also been reported [62]. In contrast, *S. frugiperda* was found to be the least susceptible of the most extensively tested insects to *B. bassiana* isolates when compared with other lepidopteran insects [63]. Hence, further studies are warranted to elucidate the mechanisms behind this low susceptibility of *S. frugiperda* larvae to the tested fungal isolates in this study. Interestingly, in contrast to the low infection rates observed, the *B. bassiana* strain caused 96.6% mortality to second instar *S. frugiperda* larvae at 1 × 10^9^ conidia ml-^1^, with a lethal time of 3.6 days [64]. Ramzi et al. [65] demonstrated that *C. suppressalis* larvae were highly susceptible to the two commercial *B. bassiana* isolates, BB1 and BB2. Resistance to fungal infection as the larvae mature may also be attributed to the composition of the larval integument that allowed for effective penetration of the fungus, resulting in increased mortality in early larval instars [66]. Meekes [67] reported that molting may be the reason for loss of inoculum and hence lower chances of fungal infection, although molting does not always result in the prevention of fungal infection. Furthermore, the low pathogenicity of fungal isolates could also be attributed to host–pathogen interactions between these isolates and *S. frugiperda* larvae, such as efficient attachment of conidia to the integument, negative impacts of integument composition with penetration tube of the fungi, and immune responses of the larvae toward conidia [52]. All these phenomena require detailed experiments to precisely elucidate the mechanism.

Insects are especially vulnerable to microbial infection during the egg development stage due to their immobility [68]. Insect eggs require great amounts of nutrients for their development until hatching; therefore, eggs are the most targetable immature stage by pathogenic microorganisms [69,70]. The results of the present study showed that the eggs of *S. frugiperda* were highly susceptible to the tested entomopathogenic fungal isolates. In line with the present study, *B. bassiana* isolates Bb39, Bb23, Bb9, Bb40, Bb19, and Bb21 showed 92, 89.2, 87.6, 82.8, 58, and 38% FAW egg mortality, respectively, at 1 × 10^8^ conidia mL^−1^ [71]. *Beauveria bassiana* isolates BbSA-1, BbSA-2, and BbSA-3 caused 100% egg mortality to the red palm weevil, *Rhynchophorus ferrugineus* (Olivier) (Coleoptera: Curculionidae) [72]. The eggs of *S. fruigiperda* were moderately susceptible to *B. bassiana* isolates ICIPE 621 and ICIPE 35, causing 51 and 29.5% egg mortalities, respectively [36]. Al-Kherb [73] also reported ovicidal activity of *B. bassiana* to the beet armyworm, *Spodoptera exigua* (Hübner) (Lepidoptera: Noctuidae), causing 50% egg mortality. Bahar et al. [58] reported that *Cladosporium* isolate RM16 caused 64% egg mortality in *Helicoverpa armigera* (Hübner) (Lepidoptera: Noctuidae). Anand and Tiwary [74] reported 100% mortality of unscaled eggs of *S. litura* due to infection with *Aspergillus* sp., while *P. citrinum* caused 2.5% egg mortality to congregating fireflies, *Pteroptyx bearni* (Coleoptera: Lampyridae) [75]. Virulence associated with *B. bassiana* to the egg stage has been previously reported for other lepidopteran insects, such as 89–100% egg mortality of the bean pod borer, *Maruca vitrata* (Fabricius) (Lepidoptera: Crambidae) [76], 27.4–96.9% egg mortality of the coffee leafminer, *Perileucoptera coffeella* (Guérin-Meneville) (Lepidoptera: Lyonetiidae) [77], and 63% egg mortality of the potato tuber moth, *Phthorimaea operculella* (Zeller) (Lepidoptera: Gelechiidae) [78]. Histopathological studies on the infection of insect eggs by EPFs revealed successful adhesion, germination, and penetration into eggs within 24 h postinoculation in the red spider mite, *Tetranychus urticae* (Koch) [79] and within 6 h postinoculation in the tomato leafminer, *Tuta absoluta* (povolny) [80], and intense extrusion of mycelium covering the eggs was observed in *T. absolute* 72 h postinoculation. In a previous report, some fungal isolates were highly virulent to neonate larvae; for example, *B. bassiana* isolates caused 53.9 to 83.9% neonate mortality to *S. fruigiperda* [36]. Entomopathogenic fungi can infect insects at any stage of their life, although not all host stages are equally susceptible to pathogen infection [38].

*Beauveria bassiana* ZK-5 and *P. citrinum* CTD-24 caused the highest cumulative mortality to eggs and neonates of *S. frugiperda* in the present study. Our findings are consistent with those of Akutse et al. [36], who found that *B. bassiana* ICIPE 621 caused a cumulative mortality of 87.5% to eggs and neonates of *S. frugiperda*. Gui et al. [59] found that *Cladosporium aphidis* fungal isolates were highly virulent to various aphid species, causing a cumulative mortality of 80% within 8 days at 1 × 10^8^ spores mL^−1^.

The considerable reduction in food consumption by insects has been attributed to the production of toxic substances by EPFs inside the host that lead to mechanical disruption in insect structural integrity [81]. *Beauveria bassiana* reduced the food consumption of the Mediterranean corn stalk borer, *Sesamia nonagrioides* (Lepidoptera: Noctuidae), by 50% at the highest conidial concentrations [82]. In previous studies, a reduction in feeding due to infection of *B. bassiana* fungus from other insect species has also been reported, such as in the Colorado potato beetle, *Leptinotarsa decemlineata* (Say) (Coleoptera: Chrysomelidae), the bean flower thrip, *Megalurothrips sjostedti* (Trybom) (Thysanoptera: Thripidae), the spotted stem borer, *C. partellus* (Swinhoe) (Lepidoptera: Crambidae), the greenish silk-moth, *Ocinara varians* (Walker) (Lepidoptera: Bombycidae), and the pea leaf miner, *Liriomyza huidobrensis* (LIRIHU) (Diptera: Agromyzidae) [34,83,84,85]. Tefera and Pringle [81] observed that *B. bassiana* significantly reduced the food consumption of *C. partellus* larvae.

In contrast, Cheung and Grula [86] reported no significant reduction in feeding for the corn earworm larvae, *Helicoverpa zea,* infected with *B. bassiana* before death. The reduction in food consumption by infected insects due to fungi is one of the important factors in host mortality, which indicates fungal pathogen virulence and needs to be further investigated to determine the level of pathogenicity or antifeedant effects [87]. *Beauveria bassiana* caused a 60% reduction in food consumption to the long-horned grasshoppers, *Uvarovistia zebra* (Uvarov) (Orthoptera: Tettigoniidae), at a concentration of 5 × 10^6^ spores mL^−1^ [88].

## 5. Conclusions

This is the first report on the use of local Chinese fungal isolates to infect *S. frugiperda* eggs, neonates, and larvae after pest invasion. The low susceptibility of sixth instar *S. frugiperda* larvae to the tested entomopathogenic fungal isolates could be a characteristic of the fungal strains tested or perhaps due to genetic diversity and phenotypic differences between *S. frugiperda* populations. This is also the first report on the impact of EPFs on the feeding efficacy of *S. frugiperda*, as effected larvae exhibited slowed feeding efficacy, leading to disruptions in larval physiology and ultimately leading to larval mortality (antifeedant effects). Two of the fungal isolates, *C. tennuisimum* and *B. bassiana,* significantly reduced the feeding performance of *S. frugiperda* larvae via indirect pathways of the fungi (different from direct contact effects). Further studies are warranted to evaluate the biopesticidal efficacy of these potent EPF isolates under field conditions before their integration into sustainable management of *S. frugiperda*.

## Figures and Tables

**Table 1:** Identity of entomopathogenic fungal isolates (EPFs) tested against eggs, larvae, and feeding performance of *S. frugiperda*.

Fungal Species	Isolates	Origin Host	Location (Country)	Isolation (Year)
*Aspergillus* sp.	BM-3	*Bombyx mori* (Lepidoptera: Bombycidae)	Guangdong (China)	2020
*Aspergillus* sp.	SE-2-1	*Trachymela sloanei* (Coleoptera: Chrysomelidae)	Guangdong (China)	2020
*C. tenuissimum*	SE-10	*Trachymela sloanei* (Coleoptera: Chrysomelidae)	Guangdong (China)	2020
*P. citrinum*	CTD-24	*Spodoptera frugiperda* (Lepidoptera: Noctuidae)	Guangdong (China)	2019
*B. bassiana*	ZK-5	*Helicoverpa zea* (Lepidoptera: Noctuidae)	Chaozhou (China)	2015

**Table 2:** Germination rates of the entomopathogenic fungal isolates used in the study.

Fungal Species	Isolates	Concentration (Conidia mL^−1^)		
		1 × 10^6^	1 × 10^7^	1 × 10^8^
*Aspergillus* sp.	BM-3	92.7 ± 2.2	92.7 ± 2.4	94.0 ± 2.5
*Aspergillus* sp.	SE-2-1	92.3 ± 1.2	91.0 ± 1.5	93.7 ± 2.3
*C. tenuissimum*	SE-10	94.3 ± 0.9	94.0 ± 2.1	93.7 ± 1.2
*P. citrinum*	CTD-24	94.0 ± 0.6	95.3 ± 0.9	93.7 ± 0.9
*B. bassiana*	ZK-5	95.0 ± 1.0	94.7 ± 1.9	96.0 ± 0.6

**Table 3:** Effects of fungal isolates on percent mortality of the sixth instar *S. frugiperda* larvae at three different concentrations.

Conidia mL^−1^	Instar	*Aspergillus* sp.		*C. tenuissimum*	*P. citrinum*	*B. bassiana*	Control
		BM-3	SE-2-1	SE-10	CTD-24	ZK-5	CK
1 × 10^6^	1st	2.2 ± 0.3	2.2 ± 0.3	3.3 ± 0.0	3.3 ± 0.0	5.6 ± 0.7	0.0 ± 0.0
	2nd	2.2 ± 0.3	3.3 ± 0.0	3.3 ± 0.0	3.3 ± 0.0	4.4 ± 0.3	0.0 ± 0.0
	3rd	1.1 ± 0.3	2.2 ± 0.3	3.3 ± 0.0	3.3 ± 0.3	4.4 ± 0.3	0.0 ± 0.0
	4th	1.1 ± 0.3	2.2 ± 0.3	1.1 ± 0.3	2.2 ± 0.3	4.4 ± 0.3	0.0 ± 0.0
	5th	1.1 ± 0.3	2.2 ± 0.3	1.1 ± 0.3	2.2 ± 0.3	4.4 ± 0.3	0.0 ± 0.0
	6th	2.2 ± 0.3	1.1 ± 0.3	2.2 ± 0.3	2.2 ± 0.3	3.3 ± 0.0	0.0 ± 0.0
1 × 10^7^	1st	4.4 ± 0.3	5.6 ± 0.3	7.8 ± 1.3	10.0 ± 1.5	14.4 ± 2.3	1.0 ± 0.0
	2nd	3.3 ± 0.6	4.4 ± 0.3	5.6 ± 0.7	7.8 ± 0.9	10.0 ± 1.5	0.0 ± 0.0
	3rd	2.2 ± 0.3	3.3 ± 0.0	4.4 ± 0.3	5.6 ± 1.2	6.7 ± 1.0	0.0 ± 0.0
	4th	2.2 ± 0.3	3.3 ± 0.0	4.4 ± 0.3	4.4 ± 0.3	5.6 ± 0.7	0.0 ± 0.0
	5th	1.1 ± 0.3	3.3 ± 0.0	4.4 ± 0.3	4.4 ± 0.3	5.6 ± 0.7	0.0 ± 0.0
	6th	2.2 ± 0.3	3.3 ± 0.0	3.3 ± 0.0	4.4 ± 0.3	5.6 ± 0.7	0.0 ± 0.0
1 × 10^8^	1st	5.6 ± 0.3	7.8 ± 0.3	11.1 ±0.3	14.4 ± 0.9	20.0 ± 0.6	0.0 ± 0.0
	2nd	4.4 ± 0.3	6.7 ± 0.0	8.9 ± 0.7	10.0 ± 0.6	15.6 ± 0.9	0.0 ± 0.0
	3rd	3.3 ± 0.0	4.4 ± 0.3	5.6 ± 0.7	7.8 ± 0.3	10.0 ± 1.5	0.0 ± 0.0
	4th	2.2 ± 0.3	3.3 ± 0.0	4.4 ± 0.3	6.7 ± 0.6	8.9 ± 0.9	0.0 ± 0.0
	5th	1.1 ± 0.3	3.3 ± 0.0	4.4 ± 0.3	5.6 ± 0.3	7.8 ± 0.3	0.0 ± 0.0
	6th	2.2 ± 0.3	3.3 ± 0.0	3.3 ± 1.0	5.6 ± 0.9	7.8 ± 0.9	0.0 ± 0.0

No statistically significant difference was observed among treatments and control across instars, and each treatment across instars within same concentration at *p* < 0.05. (Tukey after one-way ANOVA).

**Table 4:** Effects of fungal isolates on cumulative mortalities of eggs and neonates at 1 × 10^6^ conidia mL^−1^.

Fungal Species	Isolates	Percent Mortality (%)		
		Egg	Neonate	Cumulated
*Aspergillus* sp.	BM-3	3.3 ± 0.0 b	2.3 ± 0.3 b	5.6 ± 0.3 c
*Aspergillus* sp.	SE-2-1	2.2 ± 0.3 b	3.4 ± 0.0 b	5.6± 0.3 c
*C. tenuissimum*	SE-10	25.6 ± 2.0 a	4.5 ± 0.6 b	32.2 ± 2.3 b
*P. citrinum*	CTD-24	30.6 ± 1.2 a	7.9 ± 0.3 ab	37.8 ± 0.9 ab
*B. bassiana*	ZK-5	40.0 ± 1.7 a	10.0 ± 1.2 a	52.2 ± 1.2 b
Control	CK	00.0 ± 0.0 c	00.0 ± 0.0 e	1.2 ± 0.5 e

Mean ± SE values within a column with different letters are significantly different at *p* < 0.05 using Tukey’s test.

**Table 5:** Effects of fungal isolates on cumulative mortalities of eggs and neonates at 1× 10^7^ conidia mL^−1^.

Fungal Species	Isolates	Percent Mortality (%)		
		Egg	Neonate	Cumulated
*Aspergillus* sp.	BM-3	5.6 ± 0.3 cd	4.7 ± 0.3 b	8.9 ± 0.3 cd
*Aspergillus* sp.	SE-2-1	20.0 ± 0.6 c	3.4 ± 0.0 b	24.4 ± 0.7 cd
*C. tenuissimum*	SE-10	40.0 ± 1.2 b	9.3 ± 0.7 ab	43.3 ± 1.2 b
*P. citrinum*	CTD-24	50.0 ± 1.7 b	15.6 ± 0.9 ab	53.3 ± 1.7 b
*B. bassiana*	ZK-5	70.0 ± 1.5 a	25.9 ± 0.9 a	73.3 ± 1.5 a
Control	CK	00.0 ± 0.0 e	00.0 ± 0.0 e	1.1 ± 0.7 e

Mean ± SE values within a column with different letters are significantly different at *p* < 0.05 using Tukey’s test.

**Table 6:** Effects of fungal isolates on cumulative mortalities of eggs and neonates at 1 × 10^8^ conidia mL^−1^.

Fungal Species	Isolates	Percent Mortality (%)		
		Egg	Neonate	Cumulated
*Aspergillus* sp.	BM-3	25.6 ± 0.9 c	7.5 ± 0.3 b	31.1 ± 1.2 c
*Aspergillus* sp.	SE-2-1	23.3 ± 0.6 c	8.7 ± 0.6 b	30.0 ± 1.0 c
*C. tenuissimum*	SE-10	55.6 ± 0.9 b	17.5 ± 0.3 b	63.3 ± 0.6 b
*P. citrinum*	CTD-24	75.6 ± 1.8 a	32.0 ± 0.3 ab	83.3 ± 1.7 a
*B. bassiana*	ZK-5	85.6 ± 1.5 a	54.3 ± 0.3 a	93.3 ± 1.2 a
Control	CK	00.0 ± 0.0 d	00.0 ± 0.0 c	1.5 ± 0.3 d

Mean ± SE values within a column with different letters are significantly different at *p* < 0.05 using Tukey’s test.

**Table 7:** Effects of fungal isolates on feeding performance of the sixth instar *S. frugiperda* larvae at 1 × 10^6^ conidia mL^−1^.

Fungal Species	Isolates	Feeding Reduction (%)					
		1st Instar	2nd Instar	3rd Instar	4th Instar	5th Instar	6th Instar
*Aspergillus* sp.	BM-3	7.1 ± 0.6 cd	6.9 ± 0.3 bc	6.7 ± 0.3 de	5.0 ± 0.6 b	4.4 ± 1.2 bc	4.2 ± 1.5 bc
*Aspergillus* sp.	SE-2-1	11.9 ± 0.3 bcd	9.7 ± 0.3 bc	8.3 ± 0.3 cd	7.2 ± 0.7 b	6.7 ± 0.6 abc	5.8 ± 1.2 bc
*C. tenuissimum*	SE-10	19.0 ± 0.3 bc	18.1 ± 0.3 ab	15.0 ± 0.6 bc	9.4 ± 0.7 ab	9.6 ± 0.9 ab	9.2 ± 0.6 ab
*P. citrinum*	CTD-24	23.8 ± 0.3 ab	22.2 ± 1.5 ab	20.8 ± 0.9 b	11.7 ± 0.6 a	11.1 ± 2.9 ab	8.9 ± 1.3 ab
*B. bassiana*	ZK-5	33.3 ± 0.3 a	31.9 ± 1.3 a	29.2 ± 1.2 a	12.8 ± 0.3 a	13.7 ± 1.2 a	12.2 ± 0.9 a
Control	CK	0.0 ± 0.0 d	0.0 ± 0.0 c	0.0 ± 0.0 e	0.0 ± 0.0 c	0.0 ± 0.0 c	0.0 ± 0.0 d

Mean ± SE values within a column not sharing a common letter are significantly different at *p* < 0.05 using Tukey’s test.

**Table 8:** Effects of fungal isolates on feeding performance of sixth instar *S. frugiperda* larvae at 1 × 10^7^ conidia mL^−1^.

**Fungal Species**	**Isolates**	**Feeding Reduction (%)**					
		**1st Instar**	**2nd Instar**	**3rd Instar**	**4th Instar**	**5th Instar**	**6th Instar**
*Aspergillus* sp.	BM-3	21.4 ± 0.0 c	25.0 ± 0.6 c	17.5 ± 0.6 d	12.2 ± 1.2 d	10.0 ± 2.0 d	6.1 ± 1.5 d
*Aspergillus* sp.	SE-2-1	33.3 ± 0.3 c	29.2 ± 0.6 c	25.8 ± 0.3 c	23.3 ± 0.6 c	19.3 ± 0.7 c	13.6 ± 1.5 c
*C. tenuissimum*	SE-10	54.8 ± 1.5 b	51.4 ± 0.9 b	45.0 ± 1.2 b	39.4 ± 1.2 ab	34.1 ± 1.2 b	21.9 ± 1.8 b
*P. citrinum*	CTD-24	57.1 ± 0.0 b	55.6 ± 0.9 b	48.3 ± 0.7 b	37.2 ± 0.7 b	33.0 ± 0.3 b	22.5 ± 1.0 b
*B. bassiana*	ZK-5	78.6 ± 0.0 a	75.0 ± 0.6 a	66.7 ± 0.3 a	46.1 ± 0.9 a	42.2 ± 0.6 a	38.6 ± 2.0 a
Control	CK	0.0 ± 0.0 c	0.0 ± 0.0 e	0.0 ± 0.0 f	0.0 ± 0.0 d	0.0 ± 0.0 d	0.0 ± 0.0 d

Mean ± SE values within a column not sharing a common letter are significantly different at *p* < 0.05 using Tukey’s test.

**Table 9:** Effects of fungal isolates on feeding performance of the sixth instar *S. frugiperda* larvae at 1 × 10^8^ conidia mL^−1^.

Fungal Species	Isolates	Feeding Reduction (%)					
		1st Instar	2nd Instar	3rd Instar	4th Instar	5th Instar	6th Instar
*Aspergillus* sp.	BM-3	11.9 ± 0.3 c	9.7 ± 0.3 d	8.3 ± 0.3 e	6.1 ± 0.7 c	5.9 ± 0.7 c	4.4 ± 1.2 c
*Aspergillus* sp.	SE-2-1	19.0 ± 0.3 bc	18.1 ± 0.7 c	18.3 ± 0.7 d	11.7 ± 1.2 c	9.6 ± 0.7 c	7.2 ± 1.3 c
*C. tenuissimum*	SE-10	42.9 ± 0.6 ab	40.3 ± 0.3 b	37.5 ± 0.6 c	20.0 ± 0.6 b	17.0 ± 0.9 b	14.2 ± 1.2 b
*P. citrinum*	CTD-24	47.6 ± 0.9 a	44.4 ± 0.3 b	42.5 ± 0.0 b	22.8 ± 0.9 b	20.7 ± 1.5 b	18.6 ± 1.5 a
*B. bassiana*	ZK-5	59.5 ± 1.3 a	55.6 ± 0.3 a	50.8 ± 0.3 a	31.1 ± 0.3 a	27.4 ± 0.3 a	21.7 ± 0.0 a
Control	CK	0.0 ± 0.0 c	0.0 ± 0.0 e	0.0 ± 0.0 f	0.0 ± 0.0 d	0.0 ± 0.0 d	0.0 ± 0.0 d

Mean ± SE values within a column not sharing a common letter are significantly different at *p* < 0.05 using Tukey’s test.
